# Cycle length and COD/N ratio determine properties of aerobic granules treating high-nitrogen wastewater

**DOI:** 10.1007/s00449-013-1102-4

**Published:** 2013-12-10

**Authors:** Agnieszka Cydzik-Kwiatkowska, Katarzyna Bernat, Magdalena Zielińska, Irena Wojnowska-Baryła

**Affiliations:** Department of Environmental Biotechnology, University of Warmia and Mazury in Olsztyn, Słoneczna Str. 45G, 10-709 Olsztyn, Poland

**Keywords:** Aerobic granular sludge morphology, *Y*_obs_, EPS, Hydrophobicity, Anaerobic digester supernatant

## Abstract

Aerobic granule characteristic in sequencing batch reactors treating high-nitrogen digester supernatant was investigated at cycle lengths (*t*) of 6, 8 and 12 h with the COD/N ratios in the influent of 4.5 and 2.3. The biomass production (*Y*_obs_) correlated with the extracellular polymeric substances (EPS) in grams per COD removed. Denitrification efficiency significantly decreased as the amount of EPS in biomass increased, suggesting that organic assimilation in EPS hampers nitrogen removal. Granule hydrophobicity was highest at *t* of 8 h; the *t* has to be long enough to remove pollutants, but not so long that excessive biomass starvation causes extracellular protein consumption that decreases hydrophobicity. At a given *t*, reducing the COD/N ratio improved hydrophobicity that stimulates cell aggregation. At *t* of 6 h and the COD/N ratio of 2.3, the dominance of 0.5–1.0 mm granules favored simultaneous nitrification and denitrification and resulted in the highest nitrogen removal.

**Electronic supplementary material:**

The online version of this article (doi:10.1007/s00449-013-1102-4) contains supplementary material, which is available to authorized users.

## Introduction

In recent years, intensive research has been carried out on the immobilization of microorganisms in aerobic conditions that results in granular sludge formation. Granular biomass is more tightly packed with microorganisms, settles faster and is more resistant to high pollutant loads than activated sludge. The main question that needs to be addressed when processing wastewater with granular sludge is how to choose the operational parameters that ensure the quality of the effluent and cultivation of highly active biomass consisting of mature granules with very good settling properties.

**Fig. 1:**
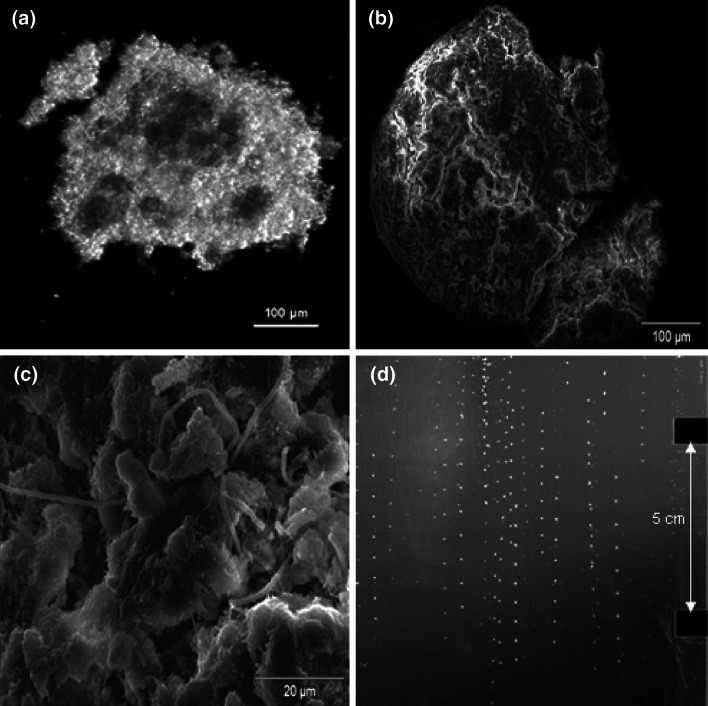
Photo of granule from light microscope (**a**, inversed), SEM (**b**, **c**), and during the free-settling test procedure (**d**)

The size of the granules results from both growth of microorganisms and hydrodynamic shearing forces in the reactor [1, 2]. Compact granules with a high density are both more resistant to decay and possess very good settling ability, expressed by sludge volumetric index (SVI). The morphology of the granules can be determined using sieve analysis, microscopic observation and free-settling tests (Fig. 1d) [3–5].

The spatial structure of microorganisms formed in wastewater treatment systems results from the development of a network of connections between the individual cells and extracellular polymeric substances (EPS) produced by the bacteria [6]. EPS excretion is a product of environmental conditions in the reactor and the availability of substrates. Proteins and polysaccharides in a form of colloids are major constituents of EPS in granules and decide about the biomass structure and properties, such as hydrophobicity [7]. EPS create a buffering layer for microorganisms against the unfavorable environment, favor organic pollutant absorption and provide a carbon and energy source during the bacterial starvation phase, but the large amount of soluble and bounded EPS negatively affects the settling and dewatering properties of sludge [8].

The morphology of the granular biomass determines the type of processes in the reactor. In loosely bounded activated sludge flocs, nitrogen removal results mainly from heterotrophic denitrification, preceded by autotrophic nitrification. Mature aerobic granules have a dense, multi-layer structure. Different substrate and oxygen concentrations in the layers of the granules allow microorganisms responsible for the oxidation of ammonia nitrogen and reduction of its oxidized forms to co-exist in the biomass, which favors the SND in the reactor. The wealth of micro-environments results in high species diversity and improves the mechanical strength of the granules.

In large granules, anoxic and anaerobic conditions occur inside the granule structure, which enable denitrification and phosphorus removal, but on the other hand, the share of anaerobic and anoxic zones in relation to aerobic zones is high, which may hamper nitrification. Liu and Tay [9] reported that the granule diameter determines specific oxygen uptake rate because of the mass transfer limitation in granules. It can therefore be concluded that the diameter of granular sludge is one of the most important variables influencing the metabolic activity of biomass and the efficiency of the treatment.

Operational parameters of a GSBR should be selected in a way that enables the formation of granules favoring the activity of microorganisms with diverse environmental demands. One of the most important operational parameters is the *t* of the GSBR. The length of the cycle must prevent biomass wash-out but ensure periodic deficiency of nutrients for microorganisms, which helps stabilize the three-dimensional structure of the granule [10]. The second important parameter that affects the strength, shape and size of the cultivated granules is the COD/N ratio of the treated wastewater. This ratio influences the balance between the percentage of autotrophic nitrifiers and heterotrophic denitrifiers in the biomass, which affects the efficiency of nitrogen removal [11].

Liu and Tay [9] observed the impact of cycle times of 1.5, 4 and 8 h on the characteristics of granular sludge in reactors fed with synthetic wastewater containing sodium acetate as a carbon source and characterized by a high COD/N ratio of 22. The authors noted that the specific COD removal rate decreased as the density and the diameter of granules increased. Research by Liu et al. [12] indicated that a decrease in the COD/N ratio from 20 to 3.3 in synthetic wastewater decreased the observed growth rate and mean size of granule diameters, and improved SVI, stability and hydrophobicity of biomass.

The evolving technology of aerobic granular sludge has great potential for application to the treatment of high-strength ammonium wastewater with a low COD/N ratio. By monitoring the physical properties of granular sludge, the state of the biomass can be assessed. This assessment allows one to predict what types of processes will occur in the biomass and to prevent the breakdown of the granules by adjustment of operational parameters. Since a low COD/N of the influent is unfavorable for denitrification, operational parameters such as cycle length of GSBR should be chosen to form granules with morphology that will favor nitrogen removal and ensure the stability of granular sludge. In the present study, the effect of GSBR cycle length and COD/N ratio in real digester supernatant on the properties of aerobic granules, including EPS production and hydrophobicity, was explored.

## Materials and methods

### Substrate

Digester supernatant was used as a substrate; it came from the “Łyna” municipal wastewater treatment plant in Olsztyn. The values of the pollutant concentrations were 802 mg COD/L, 474 mg N-NH_4_^+^/L, 570 mg N/L, 12 mg P/L. Carbonates and carbohydrates were added to the supernatant to ensure nitrification. Sodium acetate was used as an external carbon source for biomass synthesis and the electron donor for denitrification.

### Experimental organization

The experiment was conducted in three column reactors with aerobic granular sludge (GSBRs). The GSBRs had a height of 100 cm, a diameter of 10 cm and a working volume of 4.5 L. The reactors were operated by programmable logic controllers at a volumetric exchange rate of 60 %/cycle and a temperature of 26 °C. Air was supplied continuously at a superficial velocity of 0.8 cm/s. The reactors were seeded with aerobic granular sludge cultivated on synthetic wastewater (about 100 mg N/L, about 600 mg COD/L in form of sodium acetate, 12 mg P/L, and macro- and microelements).

The *t* of the GSBR were 6, 8 and 12 h. The GSBRs were operated as follows: filling (5 min), aeration (345–705 min depending on the cycle length), settling (5 min), and decantation (5 min). In series 1, the supernatant diluted with tap water in a 1:1 (v:v) proportion was fed to the GSBRs. In series 2, the reactors were supplied with undiluted supernatant. The total nitrogen concentration in the influent equaled 290 mg N/L and 570 mg N/L in series 1 and 2, respectively. In both series, sodium acetate was added to the influent to obtain the COD concentration of 1,300 mg COD/L. It resulted in COD/N ratios of the feeding of 4.5 and 2.3 in series 1 and 2, respectively. Operational parameters in experimental series are given in Table 1. Each series was abbreviated in accordance with a format in which the first term in the subscript refers to the percentage of the anaerobic sludge digester supernatant in the influent and the second indicates the *t* (e.g., *R*_50%_6h_—a GSBR working in a 6-h cycle fed with diluted supernatant from an anaerobic sludge digester).

**Table 1:** Operational parameters in the experimental series

Parameter	*R*_50%_6h_	*R*_50%_8h_	*R*_50%_12h_	*R*_100%_6h_	*R*_100%_8h_	*R*_100%_12h_
*n*	95	70	50	120	90	60
HRT, h	9.6	12.8	19.2	9.6	12.8	19.2
VLR, kg COD/(m^3^ day)	2.9	2.2	1.5	3.8	2.8	1.9
*r*_C,_ g COD/(g TSS day)	0.20	0.24	0.17	0.69	0.57	0.40
*r*_N,_ g N/(g TSS day)	0.06	0.07	0.05	0.23	0.18	0.13

*n* numbers of GSBR cycles at steady-state conditions, *VLR* volumetric loading rate, *HRT* hydraulic retention time, *r*_*C*_ organics loading, *r*_*N*_ nitrogen loading

### Analytical methods

Wastewater and biomass in the reactors were analyzed in accordance with APHA [13]. The EPS amount was measured according to Hang et al. [14]. The amount of EPS produced for a unit of COD removed (g EPS/g COD_rem_) was calculated as described by Laspidou and Rittmann [15]. Relative hydrophobicity was assessed in accordance with Chang and Lee [16]. Biomass production was expressed as *Y*_obs_ and calculated according to Klimiuk and Kulikowska [17]. The granular sludge properties were assessed on the basis of the SVI after 5 and 30 min of sedimentation (SVI_5_, SVI_30_) [13] and the free-settling test procedure [5]. Granular sludge samples were collected at the end of each experimental series. Photos were taken with an OLYMPUS camera with a resolution of 10 Mpics. 290 of the photographed granules were analyzed using the Image Tool Version 3.0 [18].

### Statistics

The differences between the two samples were determined using a *t* test for independent samples. To compare more than two samples, an RIR Tukey test was used. The relationships between individual results were determined using Pearson’s correlation coefficient. The data analysis was performed using STATISTICA 8.0 (StatSoft, USA). A value of *p* ≤ 0.05 was defined as significant.

## Results

Granule morphology and nitrogen removal efficiency were investigated during the treatment of high-nitrogen anaerobic digester supernatant by aerobic granular sludge at different *t* of GSBR and the COD/N ratios in the influent of 4.5 and 2.3. Table 2 summarizes the biomass characteristics in the experimental reactors.

**Table 2:** Biomass characteristics in the experimental series

	*R*_50%_6h_	*R*_50%_8h_	*R*_50%_12h_	*R*_100%_6h_	*R*_100%_8h_	*R*_100%_12h_
TSS, kg TSS/m^3^	15.6 ± 2.1	9.7 ± 0.9	9.4 ± 1.9	6.9 ± 1.7	6.4 ± 1.5	5.8 ± 0.7
VSS, kg VSS/m^3^	7.4 ± 0.6	5.0 ± 0.4	4.5 ± 0.7	4.1 ± 1.4	4.3 ± 1.1	4.0 ± 0.7
*Y*_obs_, g TSS/g COD_rem_	0.24	0.48	0.40	0.46	0.36	0.63
SVI_5_/SVI_30_, mL/g TSS	26 ± 15/24 ± 14	34 ± 10/32 ± 14	31 ± 10/27 ± 10	32 ± 3/28 ± 2	32 ± 7/26 ± 7	43 ± 21/34 ± 16
*D*, mm	1.9 ± 0.6	1.9 ± 0.5	1.7 ± 0.5	1.4 ± 0.4	0.9 ± 0.2	1.1 ± 0.2
*V*, mm/s	2.4 ± 1.8	2.8 ± 1.4	3.3 ± 1.9	2.5 ± 2.7	1.3 ± 0.6	1.4 ± 0.8
*V*/*D* ratio, s^−1^	1.5	1.6	0.8	3.2	2.6	1.7
*Re*	4.18 ± 4.10	4.53 ± 2.99	5.06 ± 4.9	3.25 ± 4.2	1.00 ± 0.62	1.25 ± 0.76
*ρ*_e_, kg/m^3^	1,001.0 ± 0.9	1,001.3 ± 0.9	1,002.3 ± 1.0	1,002.2 ± 2.3	1,003.3 ± 1.9	1,002.8 ± 2.2
*m*, mg	0.006 ± 0.006	0.007 ± 0.004	0.007 ± 0.007	0.004 ± 0.006	0.002 ± 0.001	0.002 ± 0.001
Fractal dimension	3.0 ± 0.9	2.9 ± 0.7	3.3 ± 0.2	2.9 ± 0.1	2.8 ± 0.1	2.9 ± 0.1

*TSS* total suspended solids, *VSS* volatile suspended solids, *SVI*_*5*_ */SVI*_*30*_ sludge volume index after 5 min of sedimentation/sludge volume index after 30 min of sedimentation, *V* settling velocity, *ρ*_*e*_ density of granule with water, *m* mass of the granule, *D* granule diameter, *Y*_*obs*_ biomass yield, *Re* Reynolds number

The granules had a spherical shape (Fig. 1a, b). Their surface was covered with a network of pores and channels enabling the diffusion of substrates to the interior of the granules (Fig. 1b). To a higher biomass concentration in the reactors undiluted supernatant was fed (Table 1). The percentages of the organic fraction in biomass (VSS) were about 50 and 70 % of dry matter during the treatment of diluted and undiluted anaerobic digester supernatant, respectively. A detailed physical characterization of the granules was made by analyzing the granular sludge photographs. The settling velocity (*V*), the density of granules (*ρ*_e_), the granule diameter (*D*), fractal dimension, and mass (*m*) were determined.

The fractal dimension of granular sludge, which shows the way the microorganisms are packed in the three-dimensional biomass structure, was high. Microorganisms of different morphologies (cocci, rods, filamentous bacteria) were densely packed on the surface of granules (Fig. 1c). The granule *ρ*_e_ of the hydrated granules ranged from 1,001.0 to 1,003.3 kg/m^3^ and was close to the density of water. The *ρ*_e_ negatively correlated with its *D* (*R* = −0.958).

Regardless of the composition of the wastewater fed to the GSBR, the *D* of the granules decreased as *t* increased from 6 to 12 h. This trend was less marked in the reactors fed with the diluted supernatant; *D* varied from 1.9 ± 0.6 mm in *R*_50%_6h_ to 1.7 ± 0.5 % in *R*_50%_12h_. In the reactors fed with undiluted supernatant, the diameter of the granules significantly decreased as *t* increased.

The *m* of granules at a particular *t* was significantly higher in the reactors fed with diluted supernatant than in the reactors fed with undiluted supernatant (*t* = 4.59, *p* = 0.010). The granules with the highest *m* were obtained in *R*_50%_12h_. In case of the reactors fed with undiluted supernatant, granules with *m* up to 0.03 mg were cultivated only in *R*_100%_6h_. Small *D* in the order of 0.47–1.57 mm was noted in *R*_100%_12h_ and *R*_100%_8h_ and *m* was up to 0.01 mg. The average weight of granules in the reactor was positively correlated with its *D* (*R* = 0.921). The relationship between *m* and *D* was clearly marked during the treatment of diluted supernatant; a change in *D* explained 46–86 % of the change in their *m* (Supplementary Material, Fig. 1S). There was no strong correlation during the treatment of undiluted supernatant.

**Fig. 2:**
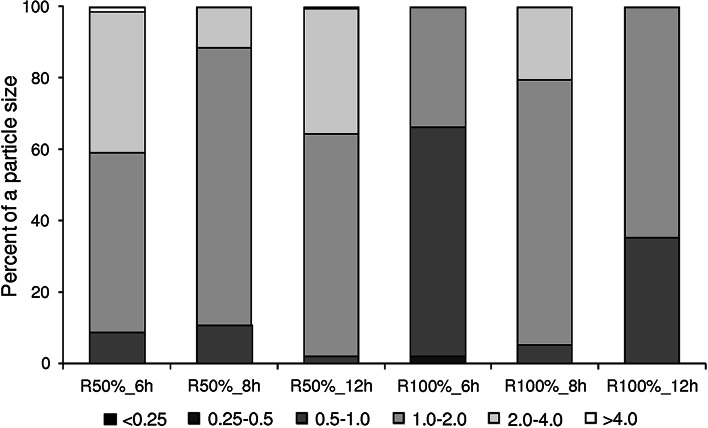
The distribution of granule particle sizes (in mm)

The *V* of granules varied widely from 0.01 to 8.00 mm/s in the reactors fed with diluted supernatant. A wide range of *V* was also noted in *R*_100%_6h_. Both *V* and *m* varied in a narrow range in the two remaining reactors fed with undiluted supernatant. As shown in Fig. 2S (Supplementary Material), there was a strong positive correlation between *m* and *V* of granules under experimental conditions (determination coefficients of 0.850–0.938). In reactors fed with undiluted supernatant, a unitary change in *m* caused a higher increase in *V* than in reactors fed with diluted supernatant.

The *V*/*D* ratio ranged from 0.8–1.6 s^−1^ during the treatment of diluted supernatant. The higher values of this ratio were recorded during the treatment of undiluted supernatant (Table 1). The best *V*/*D* ratio was found in biomass from the reactor operated at *t* of 6 h. Our tests showed that the correlation between *V* and *D* was very weak. This indicates that *D* determined *V* of the biomass only to a small extent and that *V* largely depended on *m* and *ρ*_e_ of the granules.

Granules were divided into six size classes, covering a range from <0.25 to >4.0 mm (Fig. 2). The distribution of granule sizes fitted to normal distribution. Most of the granules had *D* in the range 0.5–4.5 mm. In all reactors, except *R*_100_6h%_, the share of granules with 1–2 mm diameter was the largest. Their percentages were highest in the reactors operated at *t* of 8 h: 77.8 and 74.4 % for *R*_50%_8h_ and *R*_100%_8h_, respectively. A large number of granules with *D* > 2 mm were observed in the reactors fed with diluted supernatant working at *t* of 6 and 12 h (the cumulative percentages were 42 and 35 %, respectively). Small granules with *D* in the range 0.5–1 mm were numerous in the reactors fed with undiluted supernatant working at *t* of 6 and 12 h. In *R*_100%_6h_, these granules accounted for as much as 65 % of all granules.

The SVI is the variable that describes the settling properties of biomass. The granules cultivated in the reactor fed with diluted supernatant had the SVI in the range of 24–34 mL/g TSS; the SVI_30_ was about 8–13 % lower than the SVI_5_. In the reactors fed with undiluted supernatant, SVI_5_ values were higher and ranged from 26 to 43 mL/g TSS; after half an hour of settling, SVI_30_ decreased by 15–25 %. Both SVIs were correlated with the *Y*_obs_ (*R* = 0.875).

**Fig. 3:**
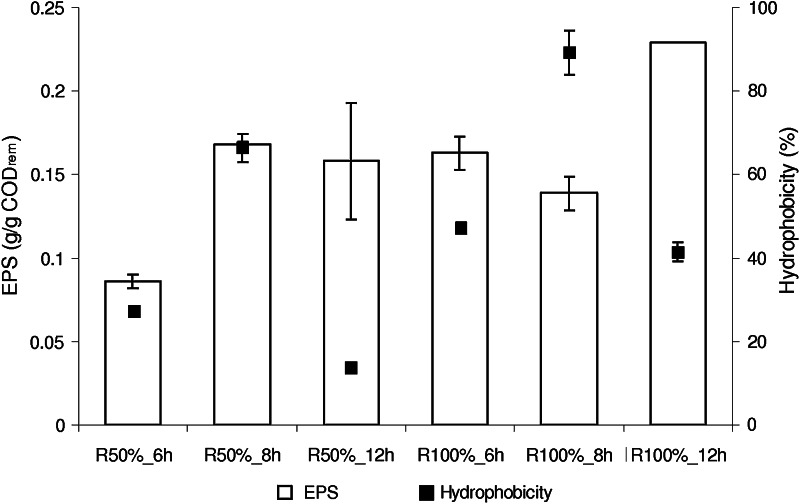
EPS production and hydrophobicity in the experimental series

The amount of EPS per unit of biomass was similar in all reactors and averaged 360 mg EPS/g TSS. This variable was negatively correlated with denitrification efficiency (*R* = −0.861) and nitrogen removal (*R* = −0.822). The highest amount of EPS per unit of COD removed was produced in *R*_100%_12h_ (Fig. 3)—0.23 ± 0.00 g EPS/g COD_rem_. The lowest amount of EPS per unit of COD removed was produced in *R*_50%_6h_—0.09 ± 0.00 g EPS/g COD_rem_. There was a strong correlation between the amount of EPS per unit of COD removed and the *Y*_obs_ (*R* = 0.994).

The highest hydrophobicity was found in the biomass from the GSBRs operated at *t* of 8 h: 66.4 ± 3.4% and 89.3 ± 5.3 % for *R*_50%_8h_ and *R*_100%_8h_, respectively. Hydrophobicity was significantly higher for a particular *t* in the reactors fed with undiluted supernatant [the difference between the averages, *p* = 0.008 (*t* = 6 h), *p* = 0.014 (*t* = 8 h), *p* = 0.002 (*t* = 12 h)].

The operational parameters of GSBRs and the resulting morphology of the granular sludge ensured efficient pollutant removal. Detailed characteristics of GSBRs operational performances were presented in [19]. Ammonium was completely oxidized in all reactors. During the purification of both diluted and undiluted supernatant, regardless of the *t* of the GSBR, the nitrite accumulation in the effluent was observed. In the reactors with granular sludge fed with diluted supernatant, 43 ± 17, 48 ± 18 and 25 ± 12 mg N/(L cycle) were denitrified at *t* of 6, 8 and 12 h, respectively. In the reactors fed with undiluted digester supernatant, significantly more nitrogen was denitrified in the GSBR cycle than in the reactors fed with diluted anaerobic digester supernatant (*t* = 3.95, *p* = 0.002) and equaled 95 ± 27, 74 ± 37 and 75 ± 28 mg N/(L cycle) at *t* of 6, 8 and 12 h, respectively. In GSBRs, the COD removal efficiency varied from 61 ± 13 to 83 ± 8%; the lower efficiencies were noted at the COD/N ratio of 2.3. In both experimental series, *t* did not significantly affect the removal efficiency of carbon compounds.

## Discussion

The COD/N ratio in the influent and the *t* of the GSBR cycle determined the morphology and properties of the granules and affected the amount of nitrogen removed in the reactor during the treatment of digester supernatant. The largest amount of nitrogen was removed per day with a 6-h reactor cycle at the COD/N ratio of 2.3, in which the morphology of the granules supported simultaneous nitrification and denitrification.

The granular biomass, irrespective of the experimental conditions, had a very high fractal dimension, indicating the presence of mature granules with stable and compact structures [20]. This is shown by the settling properties of the sludge (SVI_5_ = 24–43 mL/g TSS) and the average *V* of the granules. It was observed that granular sludge concentration decreased as *t* increased. The amount of biomass at a given *t* was additionally decreased by reducing the COD/N ratio of wastewater. Our molecular results (data not presented) showed that this may have occurred because doubling the nitrogen load in the influent increased the percentage of bacteria of the first phase of nitrification and Anammox microorganisms with a low growth rate.

The size of the granules in the experiment depended on both the composition of the wastewater and the *t*. Increasing the organic carbon and nitrogen load of the biomass by introducing undiluted supernatant to the reactors significantly reduced the granule *D* when compared to that observed during the treatment of diluted supernatant. This is consistent with the observations of Kishida et al. [21] who removed nitrogen from livestock wastewater with 650 mg N/L and constant COD/N ratio of 5 in a reactor with an aerobic granular sludge. The authors initially treated tenfold diluted wastewater, then undiluted effluent was fed into the reactors, which decreased the *D* of the granular sludge from 0.4–0.5 mm to about 0.25 mm. Our study shows that at low COD/N ratios, the *D* of the granules is reduced by lengthening *t* from 6 to 12 h. At a much higher COD/N ratio of 38, this decrease in *D* with increasing *t* has previously been noted [9].

The literature indicates that, at a depth of about 800 μm, the diffusion of substrates is inhibited, which leads to bacterial cell death [4]. Therefore, the highest microbial activity per unit of biomass is noted in granules with *D* < 1,600 μm. Li and Liu [22] have shown that dissolved oxygen is the main limiting factor for granules with a size higher than 0.5 mm in reactors with an acetate concentration of 400 mg COD/L and an oxygen concentration of 8 mg O_2_/L. From this, it can be concluded that *D* should be >0.5 mm for efficient SND, and lower than 1.6 mm to prevent cell death in the inner part of the granule. The largest fraction of the granules with this desired size was observed in the reactor fed with undiluted supernatant and operated at *t* of 6 h. In this reactor, the highest amount of nitrogen was removed in denitrification both per reactor cycle [95 ± 27 mg/(L cycle)] and per day [380 mg N/(L day)].

When the COD/N ratio was reduced from 4.5 to 2.3, the denitrification efficiency was also reduced; it was calculated as the total amount of N removed in relation to the total amount of NO_x_ in the reactor. However, the total amount of nitrogen removed at a given *t* was higher. The higher amount of reduced nitrogen in series 2 may have resulted from the better availability of organic compounds due to the higher VLR. Extending the GSBR cycle, regardless of the COD/N ratio in the influent, decreased the nitrogen removal efficiency. This was most likely caused by the reduced availability of COD associated with the less frequent addition of substrate.

Our studies have shown that the *ρ*_e_ of aerobic granules was more strongly determined by the nitrogen load than the organic carbon load. Li et al. [23] observed that at an organic load of 1.5 kg COD/(m^3^ day) (COD/N = 9.6) in the reactor were 2 mm granules with a high *ρ*_e_. 4–5 mm granules with looser structure were observed at higher loads of 3.0 kg COD/(m^3^ day) and 4.5 kg COD/(m^3^ day) (COD/N of 19 and 29, respectively). In the present study, the reverse trend was observed. Despite organic VLR increasing to the values close to those in the cited work due to dosing of undiluted supernatant, an increase in the *ρ*_e_ and a decline in the *D* of the granules were observed.

Our data have shown that the use of organic compounds for EPS production is influenced by both *t* and COD/N ratio in the influent. EPS accounted for about one-third of the mass of the granules; their content in the biomass did not differ significantly in the experimental series; however, the amount of EPS produced per unit of COD removed changed. The smallest amount of EPS per COD_rem_ was recorded in *R*_50%_6h_, the highest (almost ¼ of COD was consumed to create EPS) was reported for *R*_100%_12h_. Starvation in the long *t*, a low COD/N ratio in the feed and a high-nitrogen load forced the use of organics for the production of EPS to maintain the structure of the biomass and to protect cells against the harmful effects of free ammonium and free nitrous acid.

In our research, the efficiency of denitrification negatively correlated with the amount of EPS per unit of biomass. This indicates that the binding of carbon to EPS, which is hardly accessible to microorganisms [15, 24], limits the amount of readily available COD and hampers the reduction of oxidized nitrogen compounds by bacteria. The presence of impermeable layer of EPS on the surface of the granular sludge can also clog the pores, which reduces the efficiency of mass transfer of the substrates into the granule structure [25] and further impairs denitrification.

The value of *Y*_obs_ informs about the mass of bacteria formed per mass of COD removed, taking into account the energy requirements. *Y*_obs_ is a product of the concentration of biomass in the reactor, the use of organics for denitrification and polymeric substance production, and the COD load, which is connected to the cycle length. There was no clear trend in the values of *Y*_obs_ in the experimental series. The concentration of organics in the influent was similar in both series. In the reactors treating diluted supernatant, the high biomass concentration meant that the organic loadings in these reactors were similarly low. In the reactor operated with a 6-h cycle, the highest organic utilization for denitrification was observed, hence the amount of organics for biomass growth was the lowest, which resulted in lower sludge yield (0.24 g TSS/g COD_rem_) than in the two remaining reactors (about 0.4 g TSS/g COD_rem_). In the reactors treating undiluted supernatant, the longer the cycle length, the lower were the nitrogen and organics loadings. Sludge yields were lower in the 6- and 8-h cycle reactors than in the 12-h reactor because of the high utilization of organics for nitrogen reduction in heterotrophic denitrification at the shorter cycle lengths. Our unpublished molecular data indicate that in the 6-h reactor, the abundance of Anammox bacteria was the highest. These bacteria do not use organics for nitrogen reduction; therefore, organics may have been used for biomass growth, which explains the higher value of *Y*_obs_ (0.46 g TSS/g COD_rem_) in this reactor than in the reactor operated with an 8-h cycle (0.36 g TSS/g COD_rem_). The highest biomass growth in the 12-h reactor (0.63 g TSS/g COD_rem_) resulted from the lowest organic utilization for heterotrophic denitrification, allowing the remaining organics to potentially be used for biomass synthesis.

The value of *Y*_obs_ in the experiment was determined by the production of EPS in the biomass. *Y*_obs_ for immobilized biomass is usually low [26]. The values of *Y*_obs_ recorded for our granular sludge were relatively high—up to about 0.6 g TSS/g COD_rem_. In our study, this can be explained by the very high influence of the EPS fraction, related to extracellular matter, on *Y*_obs_. The high content of EPS in granular sludge makes it difficult to draw conclusions about the differences in growth of biomass in activated and granular sludge, if this growth is understood as the synthesis of new microbial cells.

The hydrophobicity of granules is inversely correlated to the quantity of surface charge and influences their stability. Increasing cell hydrophobicity causes a decrease in the excess Gibbs energy of the surface that tightly aggregates bacteria. It had been suggested that the granular sludge surface charge and hydrophobicity are related to the proportions of EPS components (proteins/carbohydrate) as well as the species composition of the biomass—fast growing bacteria had a greater level of negative charge than slow-growing bacteria [27]. Sheng et al. [6] have connected the increase in surface charge and hydrophobicity during the formation of aerobic granular sludge with an increase in the protein/polysaccharide ratio (PN/PS)—the increase in protein content decreases the surface negative charge of cells and favors bridging between two cells.

In our study, reducing the COD/N ratio from 4.5 to 2.3 significantly increased biomass hydrophobicity by 1.7–3.0 times, irrespective of the *t* of the GSBR. Research by Durmaz and Sanin [28] has shown that a change in the ratio of carbon to nitrogen compounds increases the amount of exopolymeric proteins in relation to polysaccharides, which may explain the improvement of the hydrophobicity of aerobic granules in our experiment.

Our results indicate that *t* strongly influences the hydrophobicity of aerobic granular sludge and should be selected so as to provide sufficient time for the removal of pollutants but not to cause excessive biomass starvation. The *t* of the GSBR adopted in the present study caused the starvation period in the reactors to last for different times. Extending the *t* from 6 to 8 h, irrespective of the composition of the wastewater, increased the biomass hydrophobicity and thus favored aggregation. This can be explained by the fact that a longer starvation period in the GSBR cycle increased the hydrophobicity of bacterial cells [12, 29]. However, further extension of *t* to 12 h drastically decreased hydrophobicity. This decrease was most likely due to the changes in the composition of the EPS in the biomass during the cycle. Studies indicate that exopolymeric proteins have been used by microorganisms in aerobic starvation as a carbon and energy source for endogenous respiration. Zhu et al. [30] observed that polysaccharide content in biomass was basically stable in the cycle of a sequencing airlift reactor, whereas EPS in the form of proteins varied significantly in biomass, reaching a maximum after the depletion of organic substrate. Our data suggest that proteins were used as carbon source during starvation, which deteriorated PN/PS ratio and decreased the hydrophobicity of biomass.

## Conclusions

- In the biomass dominated by granules with the diameter of 0.5–1.0 mm, from GSBR operated at a cycle length of 6 h and the COD/N ratio of 2.3, simultaneous nitrification and denitrification were favored that resulted in the highest nitrogen removal per day.
- The efficiency of denitrification significantly decreased as the amount of EPS per unit of granular biomass increased. To maintain high simultaneous nitrification and denitrification during the treatment of wastewater with a low COD/N ratio, operational parameters should be adjusted to prevent excessive production of EPS.
- In all experimental series, the aerobic granular biomass had excellent settling properties and stability. The diameter of granules was reduced by either changing the COD/N ratio of the influent from 4.6 to 2.3 or increasing the cycle length.
- The *Y*_obs_ was inflated by the production of EPS in the biomass. Therefore, because of the high EPS content in the granules, a comparison between the microbial cell growth in granular sludge and in activated sludge may be inadequate if *Y*_obs_ is used.

## Electronic supplementary material

Below is the link to the electronic supplementary material.

Supplementary material 1 (DOC 73 kb)
