# Supplementary material for: Cycle length and COD/N ratio determine properties of aerobic granules treating high-nitrogen wastewater
Source: Bioprocess Biosyst Eng. 2013 Dec 10;37(7):1305–13. doi: 10.1007/s00449-013-1102-4 (PMC4065379; doi:10.1007/s00449-013-1102-4)
Supplement: Supplementary file 1 — Supplementary material 1 (DOC 73 kb) [file 449_2013_1102_MOESM1_ESM.doc]

**Cycle length affects properties and denitrification potential of aerobic granules treating high-nitrogen wastewater**

Agnieszka Cydzik-Kwiatkowska, Katarzyna Bernat*, Magdalena Zielińska, Irena Wojnowska-Baryła

University of Warmia and Mazury in Olsztyn, Department of Environmental Biotechnology, Słoneczna Str. 45G, 10-709 Olsztyn, Poland

* Corresponding author, e-mail: katarzyna.bernat@uwm.edu.pl, tel.: 48 89 523 41 18, fax: 48 89 523 41 31

Fig. 1S Relation between mass and diameter of granules obtained during the treatment of diluted and undiluted anaerobic digester supernatant

Fig. 2S Relation between mass and settling velocity of granules obtained during the treatment of diluted and undiluted anaerobic digester supernatant
